# QuickStats

**Published:** 2013-11-01

**Authors:** Jiaquan Xu

**Figure:**
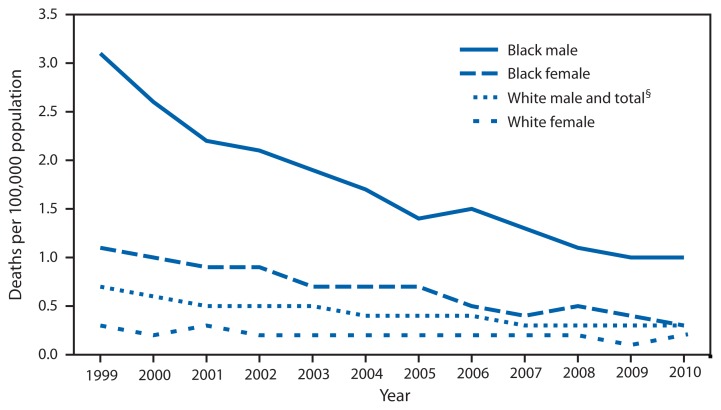
Age-Adjusted Death Rates^*^ from Tuberculosis,^†^ by Race and Sex — National Vital Statistics System, United States, 1999–2010 ^*^ The populations used for computing death rates were enumerated as of April 1 for 2000 and 2010, and estimated as of July 1 for all other years. ^†^ Includes deaths from tuberculosis as underlying and contributing causes of death, which are coded to A16–A19, according to the *International Classification of Diseases, 10th Revision*. ^§^ The death rates for 1999 – 2010 for white males matched the rates for the total population at the single-digit level. Therefore, this line represents both groups in this figure.

From 1999 to 2010, age-adjusted death rates from tuberculosis decreased 57.1%, from 0.7 to 0.3 per 100,000 population for the total U.S. population. The rate decreased 67.7% for black males, 72.7% for black females, 57.1% for white males, and 33.3% for white females. Throughout the period, the rates for black males were the highest and at least 5 times higher than the rates for white females, the group with the lowest rates.

**Source:** National Vital Statistics System. Mortality public use data files, 1999–2010. Available at http://www.cdc.gov/nchs/data_access/vitalstatsonline.htm.

